# Relevance of Anthocyanin Metabolites Generated During Digestion on Bioactivity Attributed to Intact Anthocyanins

**DOI:** 10.3390/foods13244066

**Published:** 2024-12-17

**Authors:** Claudia I. Victoria-Campos, José de Jesús Ornelas-Paz, Claudio Rios-Velasco, Saul Ruiz-Cruz, Juan Ornelas-Paz, Carmen L. Del Toro-Sánchez, Enrique Márquez-Ríos, Rogelio Calderón-Loera

**Affiliations:** 1 Facultad de Enfermería y Nutrición, Universidad Autónoma de San Luis Potosí, Niño Artillero 183, Zona Universitaria, San Luis Potosí 78240, San Luis Potosí, Mexico; claudia.victoria@uaslp.mx; 2 Centro de Investigación en Alimentación y Desarrollo A.C.-Unidad Cuauhtémoc, Av. Río Conchos S/N, Parque Industrial, Cd. Cuauhtémoc 31570, Chihuahua, Mexico; claudio.rios@ciad.mx (C.R.-V.); juanopmx@gmail.com (J.O.-P.); calderondentista@gmail.com (R.C.-L.); 3 Departamento de Investigación y Postgrado, Universidad de Sonora, Blvd. Rosales and Luis Encinas S/N, Hermosillo 83000, Sonora, Mexico; saul.ruizcruz@unison.mx (S.R.-C.); carmen.deltoro@unison.mx (C.L.D.T.-S.); enrique.marquez@unison.mx (E.M.-R.)

**Keywords:** anthocyanin metabolites, bioactivity, nutraceuticals, chemical stability, dietary pigments, flavonoids

## Abstract

Epidemiological and *In vitro* studies suggest that dietary anthocyanins in their intact form exert beneficial effects on human health. However, the potential contributions of anthocyanin metabolites to these beneficial effects have been underestimated. The objective of this review was to critically analyze the outcomes of studies concerning the formation, identification, cellular transport, and biological actions of anthocyanin metabolites generated during digestion to formulate several premises supporting the idea that these compounds largely contribute to human health. Studies performed using purified or semi-purified anthocyanins under digestion or physiological conditions were prioritized in this review. It was found that the information available about the digestive stability and metabolism of anthocyanins, as well as about their transport and deposition in human tissues has mostly been generated using plant extracts or tissues naturally containing compounds identified as anthocyanin metabolites or compounds that can serve as precursors of compounds identified as anthocyanin metabolites. This has significantly compromised the accurate identification of anthocyanin metabolites. Studies with pure or semi-purified anthocyanins are scarce in this regard. Some analytical procedures have also led to the unreliable identification and quantification of anthocyanin metabolites and, consequently, to the unreliable determination of their contribution to human health. Evidence suggests that anthocyanins are also highly metabolized in the gastrointestinal tract and transported, stored, and biologically active as their intermediary structures and final metabolites.

## 1. Introduction

Plant foods rich in anthocyanins have shown antioxidant, anticancer, antidiabetic, and anti-inflammatory effects, as well as the property to improve cognition and neuronal activities and prevent cardiovascular diseases (Table 1). The main health benefits of anthocyanins are related with cardiometabolic effects, including the decrease in blood pressure, inflammatory cytokines, reactive oxygen species, atherosclerosis, triglycerides, LDL-cholesterol, and fasting blood glucose [1,2,3,4,5,6,7,8,9,10,11]. These effects favor the anti-inflammatory activity of anthocyanins associated with their neuroprotective [12,13,14,15] and anticancer effects [16,17,18,19]. The available evidence about the protective effects of anthocyanins is controversy and there are not sufficient clinical and epidemiological trials to stand objectively these effects. *In vitro* and *in vivo* studies have demonstrated that a very low proportion of the consumed anthocyanins (less than 5%) is absorbed in intact form in the digestive tract (DT) [20,21,22,23]. This low bioavailability of intact anthocyanins does not match the high bioactivity attributed to these compounds (Table 1).

**Figure 1:**
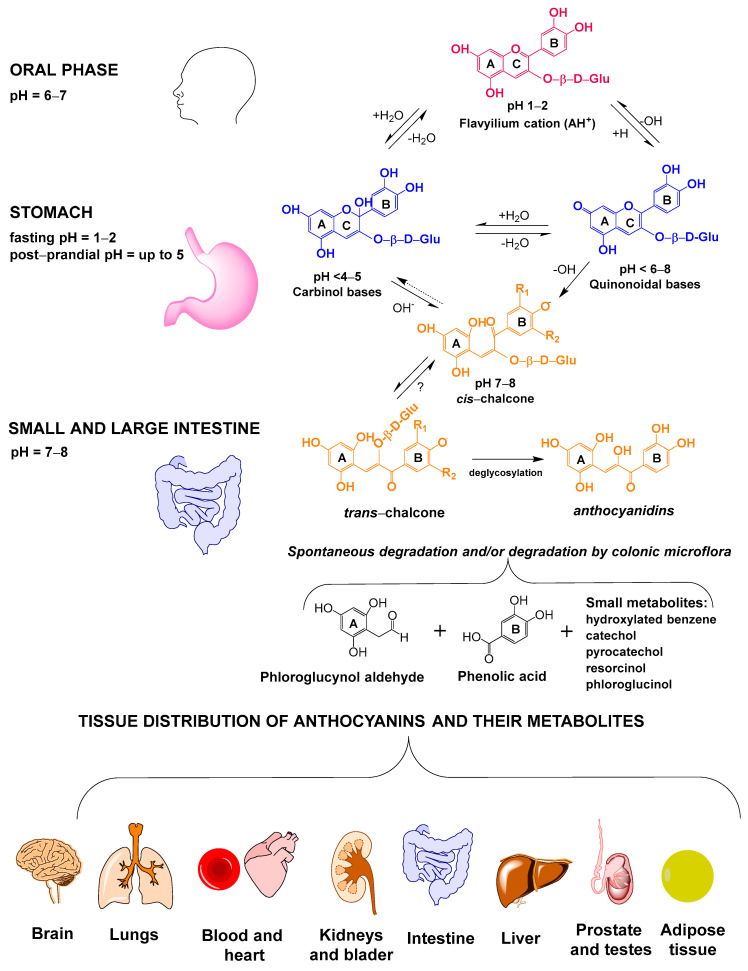
Anthocyanin transformations under physiological conditions and tissue distribution.

On the other hand, it has been demonstrated that anthocyanins are highly susceptible to chemical modification due to several factors, especially medium pH (Figure 1). Anthocyanins are exposed to acidic and alkaline conditions in the DT while circulating and deposited anthocyanins remain in an environment with a pH close to neutrality [23,24]. Thus, high quantities of anthocyanin metabolites are expected in chime and organism tissues, leading to think that anthocyanin metabolites largely contribute to the biological actions regarded to intact anthocyanins. Unfortunately, the study of anthocyanin stability in the DT and subsequent transport and bioactivity has been mostly assessed by using anthocyanin-rich plant extracts or tissues already containing compounds that have been identified as anthocyanin metabolites, making it difficult to determine the role of anthocyanin metabolites on health [24,25,26].

The existence of several anthocyanin forms in aqueous solutions has been known for decades [27,28]. Some studies have suggested that some of these forms are more active biologically than the flavylium cation, the most studied and colored anthocyanin form [29,30,31]. Unfortunately, some of these anthocyanin forms are colorless and their identification and quantification are challenging using common spectrophotometric analysis. This issue has traditionally been solved by acidifying the media to form the flavylium cation again, which can be easily detected using common analytical equipment. Several studies have suggested that this analytical strategy might lead to underestimating or overestimating the quantity of these forms and, therefore, the potential contribution of these compounds to human health [24,30,32]. This review critically analyzes the outcomes of studies concerning the formation, identification, cellular transport, and biological actions of anthocyanin metabolites generated during digestion to formulate several premises supporting the idea that these compounds largely contribute to human health. Studies performed using purified or semi-purified anthocyanins under digestion or physiological conditions were prioritized in this review without considering the publication year.

## 2. Premises

### 2.1. Premise I. Anthocyanin Structure Is Highly Influenced by pH

The anthocyanin structure possesses the characteristic flavonoid carbon skeleton, which can additionally contain monosaccharides, hydroxyl, and/or methoxyl groups [33,34]. The sugar moieties enhance anthocyanin solubility and can be acylated with the acids *p*-coumaric, caffeic, sinapic, *p*-hydroxybenzoic, malonic, and acetic. In an aqueous solution, the anthocyanins exist in equilibrium as the flavylium cation, hemiacetal, quinoidal bases, and chalcones (Figure 1) [27,28]. The flavylium cation is the most colored and abundant form in acidic media (pH 1–3). Any of its hydroxyl groups can be deprotonated at pH 4 to 6, resulting in neutral forms of anthocyanins. A second hydroxyl group may be deprotonated at neutral or alkaline pH to form negatively charged species. These negative and neutral forms are responsible for the bathochromic shift in the visible spectrum when the pH increases from 2 to 10 [33,35]. On the other hand, the nucleophilic hydroxyl ion of water may react with the C-2 of the flavylium cation and lead to the formation of carbinol pseudobase. The absence of a double bond between rings A and B in the carbinol form causes the loss of absorbance in the visible region of the light spectrum. The carbinol pseudobase may be transformed to *cis*- or *trans*-chalcones through a prototopic tautomerism due to the opening of the C-ring. The hemiacetal form and quinoidal bases are also formed from the flavylium cation as a function of pH changes and serve as chalcone precursors. Solutions with high proportions of chalcones are distinguished by pale yellow colorations (λ_max_ at 350 nm). The chalcones are predominant in slightly acidic media when there is not a substituent in the C-3, but generally, anthocyanins have a monosaccharide attached in this position [28,33]. The chalcone formation is evidenced by increased absorbance at 350 nm [33,36]. Although many other factors can promote chalcone formation (e.g., the presence of phenolic compounds and some minerals in the medium, the velocity of temperature change, and light, among others), medium pH is the most important factor leading to the formation of these anthocyanin forms [23,33,36]. It is important to indicate that the pH-mediated formation of chalcones from anthocyanins highly depends on anthocyanin structure, including the monosaccharide moiety, acylation, and other functional groups (e.g., methoxylation) [31,36]. The dependence of the anthocyanin structure on media pH must be considered in studying the stability, metabolism, transport, and accumulation of these compounds in the human body.

### 2.2. Premise II. Anthocyanin Structure Is Modified After Consumption

Anthocyanin transformation starts in the oral phase of digestion and continues during all digestive steps (Figure 1). The pH is the most important factor affecting the digestive stability of anthocyanins, and pH in the digestive medium is so variable, depending on several factors associated with subject health and ingested meals. This adds complexity to the understanding of anthocyanin metabolism. Anthocyanins are highly stable as flavylium cations at the acidic conditions in the stomach (pH 2 at fastening); however, meal composition can modify the pH of the gastric media in the range of 2 to 4, and, therefore, the anthocyanin structure [36]. Although body temperature varies slightly during the day, it can also alter the metabolism of anthocyanins, as some of these metabolic reactions are endothermic. The digestive metabolism of anthocyanins also depends on their structure. Recently, we demonstrated that the digestive stability of 3-glucoside forms of anthocyanins depended on anthocyanidin structure in the order of cyanidin > malvidin > pelargonidin [23]. The number and position of oxygenated substituents in the anthocyanidins also affect the digestive stability of anthocyanins, especially –OH and –OCH_3_ groups [23,30,37]. Glycation is another structural factor affecting the digestive stability of anthocyanins, observing high differences between 3-O-glycosides and 3,5-O-diglycosides [37]. Glycation prevents the rupture of the anthocyanin B ring [35]. We also demonstrated that the *In vitro* digestive stability of several anthocyanins depended on their glycation, decreasing in the order of glucose > rutinose > galactose [23], although other orders of protection for sugars have also been reported [30].

Anthocyanins are transformed into several metabolites under physiological conditions (Table 2 and Table 3). Kamonpatana et al. [30] found that *ex vivo* salivary incubations of cyanidin-3-glycoside induced the formation of chalcone glucosides of cyanidin, which explained up to 30% of the degradation of such compound. These chalcones were identified by LC-MS-MS, but they [30] did not provide structural information (fragment ions) for future identification of these compounds or special considerations about sample handling. Kalt et al. [32] demonstrated that humans mainly excreted blueberry anthocyanins as aglycone glucuronide forms, which had the same molecular ion as the parent anthocyanidin, suggesting that the digestion process and transport of anthocyanins in the human organism lead to different forms of the same anthocyanin, which can be finally transformed in more stable forms like *trans* and *cis* chalcones. This study also suggested that demethylation and dehydroxylation of the anthocyanin structure may occur during anthocyanin digestion, transport, and metabolism, and that such reactions may induce the interconversion of anthocyanins, with these reactions favoring the formation of pelargonidin-like structures [32]. Wu et al. [38] also reported the transformation of cyanidin-3-glucoside to its 3-O-methylated form, peonidin-3-glucoside, after consuming elderberry extracts. Fornasaro et al. [39] reported the appearance of peonidin-3-glucoside, malvidin-3-glucoside, delphinidin-3-glucoside, and perlargonidin-3-glucoside in plasma after the intravenous administration of cyanidin-3-glucoside in rats. Our research group recently observed that several pure anthocyanins were metabolized almost exclusively to chalcone glycosides during *In vitro* gastrointestinal digestion [23]. Others have also observed chalcone glycosides and chalcone pseudobase after the first hour *In vitro* gastric-intestinal and large intestine digestion of purified anthocyanins (cyanidin-3-glucoside, cyanidin-3-rutinoside and delphinidin-3-rutinoside [40]. For the identification and traceability of anthocyanin isoforms, future studies must consider their interaction with physiological ions, as our research group recently found for the first time sodium adducts of chalcones in the digestive media [23]. Until now, there have not been reports about the possible structure stabilization effects of ions on metabolites of phenolic compounds.

The identification of glucuronide, methylated, and sulfuronide conjugates of anthocyanins in urine highlights the role of UDP-glucuronosyl transferase, UDP-glucose dehydrogenase, and catechol-O-methyltransferase in the intestinal metabolism of anthocyanins [41]. Some of these reactions may also occur in the kidney and liver [41,42,43,44,45]. Płatosz et al. [46] observed 21 circulating anthocyanin metabolites (i.e., methylated, glucurono- and sulfo-conjugates of anthocyanins) in sheep fed with chokeberry anthocyanins. These changes in anthocyanin structure may alter the reactivity of anthocyanins and, therefore, their bioactivity. This type of anthocyanin metabolite has been identified in several studies, and they did not represent more than 2% of the ingested anthocyanins [38,47]. Glucuronidation reaction is possible due to the substitution of hydroxyl groups in the flavonoid-like structure of anthocyanins. The susceptibility of cyanidin to be transformed into peonidin suggests that C3′ is the most probable site for this substitution.

On the other hand, the almost instantly conversion of anthocyanins to their constitutive phenolic acids and phloroglucinol aldehyde has been demonstrated during the incubation of anthocyanins in media mimicking the gastrointestinal conditions [37,48,49,50]. Kay et al. [51] observed high degradation rates for cyanidin-3-glucoside after incubation in different buffers (phosphate buffer, DMEM, and Hank’s buffer) at 37 °C and observed that protocatechuic acid and phloroglucinol aldehyde were the final products; however, other intermediate products were also detected and subjectively/tentatively identified as C-ring open structures of cyanidin. They did not find differences between incubations with or without Caco-2 cells, highlighting the influence of digestive media on anthocyanin stability and potentially discarding the anthocyanin metabolism at the surface of the DT. Azzini et al. [49] observed that the content of 4-hydroxybenzoic acid and protocatechuic acid in plasma explained 34% to 59% of the metabolism of pelargonidin-3-glucucoside and cyanidin-3-glucoside ingested by fastened subjects. Similarly, Vitaglione et al. [50] reported that the synthesis of protocatechuic acid accounted for about 73% of the cyanidin-3-glucoside from blood orange juice ingested by fasted subjects. Deglycosylation of anthocyanins during digestion or absorption may enhance their susceptibility to degradation to phenolic acids and phloroglucinol aldehyde [49]. The *in vivo* characterization of anthocyanin degradation pathways to phenolic acids using foods is impossible because foods naturally contain a wide diversity of phenolic acids.

Additionally, Nurmi et al. [52] emphasized the possible interconversion between phenolic acids during metabolism and that the intestinal flora plays an important role in these transformations. Due to the low effect of intestinal β-glucosidases on anthocyanin structure (cytosolic β-glucosidase or lactase-phlorizin hydrolase), some studies have focused on the study of anthocyanin metabolism by intestinal microflora (Table 4), demonstrating consistently the metabolism of anthocyanins into aldehydes and their constituted phenolic acids at colonic conditions [24,25,26,53,54,55,56,57,58,59]. Keppler and Humpf [58] found an inverse correlation in the concentration of anthocyanin and the degradation products (phenolic acids and phloroglucinol aldehyde) in the first 20 min of incubation of pure anthocyanins with pig intestinal microbiota. The anthocyanin deglycosylation resulted in anthocyanidins being more unstable in alkaline conditions. The phenolic acids can be metabolized into smaller molecules, which are more stable and easily absorbable than their parent phenolic acids and anthocyanins [41]. The final anthocyanin metabolites (i.e., phenolic acids and phloroglucinol aldehyde) are not always observed in *In vitro* digestions, probably due to a higher digestion time required to achieve anthocyanin degradation or because these metabolites are not stable in a digestion medium [23,37,60]. Gamel et al. [61] did not detect anthocyanin metabolites in plasma and only detected ferulic acid in urine as the final anthocyanin metabolite of healthy humans after consumption of purple wheat crackers. However, ferulic acid could be naturally present in the tested food. Phenolic acids could also be further metabolized and used as energy sources by the gut microbiome [58] (Table 4). Sánchez-Patán et al. [59] reported dihydroxylated benzene, catechol and pyrocatechol structures presumably as phloroglucinol metabolites after fermentation of wine phenolic extracts using a human feces inoculum. Stalmach et al. [26] found pyrogallol, catechol, resorcinol, and phloroglucinol after colonic fermentation of concord grape juice; however, these metabolites are common for flavonoids with hydroxyl groups in the A ring, not only from anthocyanins.

### 2.3. Premise III. Anthocyanins and Their Metabolites Exert Beneficial Effects on Human Health

Small amounts of intact anthocyanins have been reported in tissues (bladder, kidney, liver, heart, brain) and biological fluids (blood, urine) (Figure 1 and Table 2) [39,42,62,63,64]. However, the presence of anthocyanin metabolites in several animal tissues suggests a biological role of these compounds in the human body (Table 2). Talavera et al. [45] found monoglucuronides of cyanidin and peonidin in the blood of rats chronically fed (15 d) with a diet supplemented with anthocyanin-rich extracts from blackberries (14.8 mmol per Kg diet). They [45] also reported the presence of anthocyanins in the kidney, liver, and brain of the supplemented rats, with cyanidin-3-glucoside being the most abundant in such tissues; however, the kidney and liver also contained important amounts of methylated and glucuronidated forms of cyanidin and peonidin. Kalt et al. [65] found intact anthocyanins in the liver, eye, cortex, and cerebellum of pigs fed with a diet supplemented with different quantities of blueberry powder during 30 d. The total concentration of anthocyanins in these tissues was independent of ingested doses, ranging from 300 to 700 pg/g fresh weight. They found higher proportions of malvidin-3-glucoside (48–68%) than peonidin-3-glucoside (3–13%) in cortex and eyes. Anthocyanin metabolites were not found in such a study; however, the acidic extraction from tissues could transform other anthocyanin isoforms into the flavylium cation, avoiding the identification of such isoforms. Aqil et al. [48] reported the presence of anthocyanidins in the lungs of rats supplemented with blueberry powders; however, they used the acidic extraction of anthocyanins, avoiding the identification of anthocyanin forms other than the flavylium cation.

Anthocyanin metabolites also exert protective effects on human health (Table 3). These compounds might largely be contributing to the beneficial effects attributed to intact anthocyanins (Table 1). Tavares et al. [66] demonstrated that gastric and intestinal digestion (*In vitro*) increased the protective activity of blackberry extracts against H_2_O_2_ injury in neuroblastoma cells compared to the non-digested extracts. This effect was observed even after dilution of digested extracts five times, and it was not mediated by the antioxidant activity of digested extracts. Interestingly, low doses of phenols found in digested samples also exhibited such beneficial effects, indicating that the anthocyanin metabolites may have bioactivity even at low absorption levels. However, although blackberries are rich in anthocyanins, metabolites from other polyphenols such as quercetin forms, lambertianin C, and Sanguiin H6 could contribute to the observed beneficial effects. The H_2_O_2_ production has been related to age-related diseases, especially neurodegeneration. Some plasma metabolites isolated from volunteers consuming an anthocyanin-rich juice exerted antiproliferative and anti-inflammatory effects in cultures of pancreatic cancer cells [67,68] (Table 3). In these studies, intact anthocyanins and their metabolite phloroglucinol aldehyde were identified in plasma along with phenolic compounds. The fermentation of Jaboticaba peel extracts with human feces also showed an antiproliferative effect in cultures of colon cancer cells; however, the fermentation led mainly to the formation of catechin, epicatechin, and tannin metabolites [69].

**Table 2:** Anthocyanin metabolites identified in animal and human organs and fluids.

Experimental Model	Type of Study	Identified Metabolites	Concentration/Content in Tissue/Fluids	Technique	Reference
Intravenous administration of cyanidin-3-glucoside in rats (668 nmol)	*in vivo*	Peonidin-3-glucoside, petunidin-3-glucoside, peonidin-3-glucoside, delphinidin-3 glucoside, malvidin-3-glucoside, pelargonidin-3-glucoside	Plasma (18.78–354.84 nM), kidneys (0.44–1.69 nmol/g), liver (5.64–58.61 pmol/g), brain (2.41–44.11 pmol/g)	UPLC-MS-MS	[39]
Supplementation of rats’ diet with blueberry powder (10 mg/day)	*in vivo*	Protocatechuic acid and anthocyanidins	Lungs (cyanidin 0.3 ng; <0.25 ng of peonidin, petunidin and malvidin) and plasma (from 55 to 100% of recovery of anthocyanins and anthocyanidins)	HPLC-MS	[48]
Rats fed with a diet enriched with blackberry anthocyanins (0.37 mmol/day of anthocyanins)	*in vivo*	Methylated forms of anthocyanins, monoglucuronides of anthocyanidins, cyanidin-3-glucoside	Liver (0.38 nmol/g), kidney (3.27 nmol/g), brain (0.25 nmol/g), and plasma (0.36 nmol/g)	HPLC-MS-MS	[45]
*ex vivo* incubation of different fruit extracts rich in anthocyanins with human saliva (15 nmol/0.6 mL of saliva)	*ex vivo*	Chalcone glucosides of cyanidin, protocatechuic acid, phloroglucinol	Oral cavity (chalones, protocatechuic acid and phloroglucinol aldehyde accounted up to 30%, 14.5% and 20% of the initial amount of anthocyanins, respectively)	HPLC-PDA-ESI-MS	[30]
*ex vivo* incubation of a black raspberry rinse formulation with human saliva (rinses with 10% black raspberry powder)	*ex vivo*	Aglycone cyanidin, protocatechuic acid, glucuronidated anthocyanin conjugates	Oral cavity (quantitiative data were not reported)	HPLC-MS-MS	[69]
Supplementation of humans with chockeberry extracts (721 mg)	*in vivo*	Methylated cyanidin glucuronide, intact anthocyanins	Serum (376.65 nmol × h/L within 7 h) and urine (1071.54 µg/24 h). 32.2% and 67.75% were present as parent anthocyanins and conjugated metabolites in both serum and urine	HPLC-MS-MS and NMR	[43]
Intestinal perfusion in rats with purified anthocyanins (10 µmol/L), blackberry (616 µmol/L) and bilberry (75.8 µmol/L) extracts	*In situ*	Monoglucuronides of anthocyanins	Plasma (121–198 nmol/L of cyanidin and cyanidin glucuronides; 56 nmol/L of peonidin and peonidin glucuronides)	HPLC-MS-MS	[44]
Blood from human volunteers after the consumption of 1 L of blood orange juice (71 mg of anthocyanins)	*in vivo*	Protocatechuic acid, cyanidin-3-glucoside	Plasma (0.03 and 65.7 µmol of cyanidin-3-glucoside and protocatechuic acid, respectively)	HPLC-MS-MS	[50]
Plasma from human volunteers after consumption of 300 g of fresh or stored strawberries (7.19–8.54 mg of anthocyanins)	*in vivo*	4-hydroxybenzoic acid, pelargonidin-3-glucoside, protocatechuic acid, coumaric acid	Plasma (13–54 µmol of intact pelargonidin-3-glucoside; 7.6–5.6, 0.1–0.18, and 0.45–0.64 µmol × h/L of 4-hydroxybenzoic acid, protocatechuic acid and coumaric acid, respectively)	HPLC-MS-MS	[49]
Supplementation of rats with blackberry extracts during 12 days (0.36 mmol/day)	*in vivo*	Methylated conjugates, monoglucuronide cyanidin	Blader (2.37 nmol/g), prostate (0.304 nmol/g), adipose tissue (0.099 nmol/g), heart (0.06 nmol/g), plasma (0.20 nmol/g) and testes (0.062 nmol/g)	HPLC-MS-MS	[42]

As was mentioned above, the final steps of anthocyanin’s digestive metabolism involve the scission of their structure to the constitutive phenolic acids and aldehydes. The health benefits of these anthocyanin metabolites have been studied and compared with those of intact anthocyanins (Table 3). These phenolic acids and aldehydes have higher health benefits than intact anthocyanins, although these effects are lower than those of complex fruit extracts. Phenolic acids and phloroglucinol aldehyde reduce the viability of cancer cell lines from the pancreas, small intestine, esophageal, colon, and breast [70,71,72,73,74,75,76]. Their main mechanisms involve the decrease in inflammation biomarkers, regulation of transcription factors associated with cell reproduction, and pro-apoptotic activity. Protocatechuic acid has a potent activity of neuroprotection, reducing the cytotoxicity and ROS levels, as well as the autophagic activity in Aβ_25-35_ stimulated primary hippocampal neurons [72]. Phenolic acids and aldehydes resulting from anthocyanin cleavage, however, do not explain all anthocyanin metabolism, and these metabolites are not always detected after digestion or ingestion of individual anthocyanins [23,39,44,45]. Further efforts are needed to elucidate the action mechanisms of metabolites in human health. The regulation of cellular pathways associated with inflammation response and reproduction cycle is not fully understood. These studies are needed to develop new therapies for chronic diseases or recommendations/guidelines for anthocyanin intake.

**Table 3:** Health benefits of common anthocyanin metabolites.

Compound/Source	Experimental Model	Effect	Active Metabolites	Reference
Metabolites extracted after absorption or digestion				
Metabolites isolated from plasma of volunteers consuming an anthocyanin-rich juice (330 mL/day; 311 mg of ACNs) for 28 days	*In vitro* exposure of pancreatic cancer cell (PANC-1 and AsPC- 1 lines) to anthocyanin metabolites	Plasma metabolites reduced the migration of PANC-1 cells but not of AsPC-1 cells. Metabolites reduced the ICAM-1 expression in stimulated PANC-1 cells. Metabolites inhibited the phosphorylation of NF-κB-p65 and FAK and reduced the levels of ROS in PANC-1 cells.	Intact anthocyanins, o-coumaric acid, and gut microbiota metabolites (PGA, MHPV-G, 3,4-DHPV)	[67]
Metabolites isolated from plasma of volunteers after 60 min of the ingestion of berry and bilberry juice (330 mL/day; 277.5 mg of ACNs)	*In vitro* incubation of the pancreatic cancer cell lines PANC-1 and AsPC-1 with anthocyanin metabolites	Plasma metabolites reduced the ROS generation. The cell migration of PANC-1 was significantly reduced by anthocyanin metabolites. Metabolites significantly reduced the expression of MMP-2, MMP-9, and NF-kB	Not specified	[68]
Extracts of non-digested or digested (*In vitro* gastric and pancreatic digestion) blackberries	Incubation of extracts with neuroblastoma cells	The ratio of antioxidant activity per total phenolic content was significantly higher in digested samples (content inside and outside dialysis bags). The digested extracts were more effective to reduce the H_2_O_2_-induced death of the neuroblastoma cells than non-digested extract	Dihydroxybenzoic acid, cyanidin metabolites, phenolic compounds and their metabolites	[66]
Jaboticaba peel powder extract (JPP)	JPP was fermented with human faces and incubated with HT29 colon cancer cells in a CRC 3D model	The fermented JPP had an antiproliferative effect on HT29 cells mainly after 8 and 24 h of incubation.	Metabolites of catechin, epicatechin and tannins (HHDP-digalloylglucose isomer and dihydroxyphenyl-γ-valerolactone), but not anthocyanin metabolites	[69]
Individual metabolites				
Black raspberry (BRB) or PCA	*in vivo* administration of control diet or diet enriched with 5% of BRB, 500 or 1000 ppm of PCA during 8 weeks to Apc^min/+^ mice with adenomas in the small intestine	BRB and PCA decreased the adenoma development in small intestine. BRB and 500 ppm of PCA decreased the inflammation biomarkers COX-2 and PGE_2_ Only 500 ppm of PCA increased the IFN-γ and SMAD4. Both BRB and PCA enhanced the proportion of anti-inflammatory bacteria and reduced the pro-inflammatory species	BRB and PCA	[70]
Freeze-dried black raspberries (BRBs), their isolated anthocyanins or PCA	*in vivo* administration of a diet containin 6.1% of BRBs, anthocyanins or PCA to esophageal cancer rats	All treatments reduced the esophageal tumorogenesis, expression of pentraxin-3, and inflammation biomarkes (COX-2, iNOS, p-NF-κB, and sEH) and cytokine (PTX3)	BRB had a higher activity than anthocyanins and PCA	[71]
Blueberry extracts and PCA	*in vivo* administration of BBE (150 mg/Kg 16 weeks) to APP/PSI mice. *In vitro* incubation of primary hippocampal neurons with PCA (400 µmol/L)	BBE decreased the dead and morphology deterioration of neurons, and the expression of autophagy-related proteins. It increased the layer of neurons. PCA reduced the cytotoxicity induced by Aβ_25–35_ in primary hippocampal neurons, inducing low levels of LDH and ROS. PCA decreased the autophagy proteins LC3-II/I and p62	BBE and PCA	[72]
Malvidin-3-glucoside and individual recognized anthocyanin metabolites	*In vitro* incubation of GA, MeGA and PGA at concentrations of 10 to 100 µM with Caco-2 cells	Caco-2 cell viability was not affected by malvidin-3-glucoside but decreased with the highest concentration of PGA. Cell viability decreased in a dose-dependent manner with MeGA and GA. MeGA and GA induced the DNA fragmentation. Three metabolites induced a significant arrest of the cell cycle at the G0/G1 and the apoptotic pathway was related to the activation of capase-3. Three metabolites decreased the transcription factors NF-κB, AP-1, STAT-1, and OCT-1.	The most potent metabolites were MeGA and PGA, followed by GA.	[73]
2,4,6-THBA, PCA, GA, and PGA	*In vitro* CDK and anti-proliferation assays in cancer cells (HCT-116, HT-29, Caco-2 and MDA-MB-231)	2,4,6-THBA exerted antiproliferative effects. It inhibited CDKs enzymes. GA and PGA did not exert effects	2,4,6-THBA	[74]
Black rice (BR, 30 µg/mL), cyanidin-3-glucisde, cyanidin and PCA (5 µM)	*In vitro* incubation of compounds with RAW 264.7 macrophage cells stimulated with lipopolysaccharide. *in vivo* administration of these compounds in carrageenan-induced inflammation mice	BR, cyanidin-3-glucoside, cyanidin and PCA inhibited the expression of IL-1β and TNF-α, PGE_2_, ON, COX-2 and iNOS. PCA had the highest inhibitory effect. Tested compounds inhibited the translocation of the p65 subunit of NF-κB into the nucleus of RAW 264 cells and activated the p38, ERK, and JNK pathways. Tested compounds, specially PCA, inhibited the expression of COX-2 and NF-κB activation	Mainly PCA	[75]
MeGA, GA, SA, PCA, VA, PGA, individual anthocyanins (petunidin-3 glucoside, peonidin-3-glucoside, and malvidin-3-glucoside)	*In vitro* incubation of metabolites and anthocyanins at concentrations from 10 to 1000 µM with Caco-2 cells	GA, MeGA, PGA and anthocyanin extract inhibited Caco-2 cell proliferation. All compounds increased the activity of the proapoptotic enzyme caspase-3, mainly SA, PCA, VA, and PGA	Mainly GA, MeGA, PGA	[76]

Abbreviations: CDK: cyclin dependent kinase; COX-2: cyclooxygenase-2; ERK: extracellular signal-regulated kinase; ICAM-1: inter-cellular adhesion molecule 1; FAK: focal adhesion kinase; GA: gallic acid; IFN-γ: interferon gamma; iNOS: nitric oxide synthase; JNK: c-jun NH2-terminal kinase; LC3-II: microtubule-associated light chain 3-II; LDH: lactate deshydrogenase; MMP: metalloproteinases; MeGA: methylgallic acid; NF-κB-p65: nuclear factor-kappa B; NO: nitric oxide; PCA: protocatechuic acid; PGA: phloroglucinol aldehyde; ROS: reactive oxygen species; THBA1d: 2,4,6-trihydroxybenzaldehyde; MHPV-G: 4′-hydroxy-3′-methoxyphenyl-γ-valerolactone glucuronide; PGE_2_: prostaglandin E_2_; SA: syringic acid; SMAD4: mothers against decapentaplegic homolog 4; VA: vanillic acid; 3,4-DHPV: 3′,4′-dihydroxyphenyl-γ-valerolactone; 2,4,6-THBA: 2,4,6-trihydroxybenzoic acid/phloroglucinic acid.

On the other hand, Grace et al. [77] demonstrated that the antidiabetic effect of anthocyanins from lowbush blueberries was greater than that of a polyphenol extract in mice. They [77] suggested that chalcones or hemiacetal forms of anthocyanins might be responsible for such beneficial effects; however, they did not identify anthocyanins or their metabolites. The hydroxylated chalcone glycoside formed during the *In vitro* oral digestion of cyanidin resembles the structure of other well-studied chalcone, butein (3,4,2′,4′,-tetrahydroxychalcone), which can suppress the transcription factor KF-κB in breast and pancreatic tumor cells as well as to reduce the cytokine-induced nitric oxide production in rat pancreatic β cells [30,78]. Some chalcone precursors of flavonoids have important anticarcinogenic activities by activating phase II metabolism enzymes, such as quinone reductase and glutathione S-transferase, and decreasing the activity of phase I enzymes (cytochrome P450). These activities depend on the methoxyl and hydroxyl substitution sites [29]. The bioavailability, accumulation in the body and health benefits of chalcones from anthocyanins have either not been or have scarcely been studied.

Metabolites generated from bacterial metabolism might also enhance the anthocyanin bioactivity. It has been observed that the deglycosylation of anthocyanins by oral microbiome enhances their chemoprotective properties in the oral cavity [79]. On the other hand, the constituent phenolic acids of anthocyanins may also impart health benefits. Aqil et al. [48] found protocatechuic acid in the lungs of rats supplemented with low doses of blueberry powders and suggested that the anthocyanin transformation to protocatechuic acid may be responsible for the anticarcinogenic effect of blueberry in the lungs. Protocatechuic acid also showed anti-inflammatory effects in inflamed cells (lipopolysaccharide-induced inflammation cells) and carrageenan-induced inflammation in air pouches in BALB/c mice. The effects of protocatechuic acid were comparable to those of the parent cyanidin-3-glucoside and cyanidin [75]. Protocatechuic acid has also been related to the *In vitro* induction of apoptosis in leukemia, human B lymphoma, and osteosarcoma cells [80,81]. Li et al. [72] observed that protocatechuic acid was the most abundant metabolite formed in mice fed with a blueberry extract and that it was responsible for neuroprotective effects against Alzheimer’s disease. Other phenolic acids are also potent antioxidants and act as chemo-preventive agents [82]. Mostafa et al. [67] observed anticancer effects in cell cultures for some anthocyanin metabolites isolated from human fluids. Milenkovic and Krga [83] demonstrated that anthocyanin metabolites can exert neurocognitive protection through a multi-genomic action mode.

Anthocyanins and their metabolites may exert systemic health benefits through indirect pathways such as regulating gut health and microbiome (Table 1 and Table 4). Protocatechuic acid induces Gram-negative bacteria death through oxidative mechanisms. The cyanidin-3-glucoside metabolites (e.g., protocatechuic, vanillic and ferulic acid) increase the activity of antioxidant enzymes in the intestine such as manganese-dependent superoxide dismutase and glutathione [84]. Phenolic acids also protect against colon cancer by avoiding angiogenesis and through anti-inflammatory activity [57]. To date, there is no full chemical characterization of the metabolites produced by gut microbiota for each anthocyanin. The bioavailability, accumulation, and health effects of these metabolites are not known. Further studies should explore the impact of the change in the gut microbiota composition by anthocyanin consumption and metabolism on human health.

**Table 4:** Anthocyanin metabolites generated by gut microbiota.

Tested Anthocyanins	Media	Metabolites	Reference
Anthocyanins from red wine	In a randomized, crossover, and controlled intervention trial, nine healthy subjects consumed red wine (270 mL/day), dealcoholized red wine (272 mL/day) or gin (100 mL/day) in periods of 20 days. Metabolites were monitored in urine and microbial content was evaluated in fecal samples	There was a positive correlation between the content of Bifidobacteria and syringic, *p*-coumaric, 4-hydroxybenzoic and homovanillic acid (*p* < 0.05). These metabolites are related to anthocyanins but the authors could not differentiate the influence of other polyphenols.	[25]
Lyophilised juçara pulp	Juçara pulp was incubated with human fecal inoculum	Benzoic, gallic, and syringic acid were detected after the first hour of incubation. Juçara pulp increased the production of acetate, propionate, and butyrate. *Bifidobacterium* spp., *Eubacterium rectale-Clostridium coccoides* group, and *Bacteroides* spp.-*Prevotella* group increased significantly.	[56]
Bilberry extracts	Healthy and ileostomy participants (*n* = 10) consumed 10 g of bilberry extracts (4.95 mmol of anthocyanins). The content of anthocyanins and their metabolites was determined in ileostomy fluids, plasma, and urine	Healthy subjects had higher concentrations of anthocyanins and their metabolites in plasma and urine than in ileostomy participants, suggesting a significant absorption in the colon. Anthocyanins were found as glucuronides in plasma, but also syringic acid (45.0%) and vanillic acid (24.6%). The main metabolites in ileostomy fluids were gallic acid (35.4%), protocatechuic acid (31.3%), and phloroglucinol aldehyde (16.7%). In urine, the main metabolites of anthocyanins were syringic acid (14.1%), protocatechuic acid (10.9%), gallic acid (9.9%), and traces of anthocyanin glucuronides.	[24]
Wine phenolic extract	The phenolic extract was incubated in a fecal batch-culture fermentation system using feces from healthy volunteers	Malvidin-3-glucoside, peonidin-3-glucoside and cyanidin-3-glucoside were largely degraded within the first 5 h, but almost 30 h of fermentation were required for their complete degradation. Dihydroxylated benzene, catechol, and pyrocatechol were progressively formed during fermentation, presumably as degradation products of phloroglucinol, which was not detected. Syringin, protocatechuic, and vanillic acid were also detected within the first 2 h of fermentation.	[59]
Concord grape juice	Juice was incubated in a model of colonic fermentation with a human fecal inoculum	The contents of 3,4,hihydroxybenzoic acid, gallic acid and small metabolites (pyrogallol, catechol, resorcinol, and phloroglucinol) increased. The authors could not distinguish anthocyanin metabolites.	[26]
Anthocyanidins (cyanidin, malvidin, pelargonidin, peonidin, delphinidin) and anthocyanins (cyanidin-3-glucoside, malvidin-3-glucoside)	Anthocyanins were incubated with human fecal microbiota samples under anaerobic conditions	90% of the anthocyanins were degraded within the first 2 h of incubation. Malvidin-3-glcuoside and peonidin-3-glucoside degraded to syringic and vanillic acid. Protocatechuic acid was generated from cyanidin-3-glucoside. Pelargonidin-3-sophoroside-5-glucoside was degraded into pelargonidin-glucoside-sophoroside and pelargonidin-sophoroside, and then, to the end products 4-hydroxybenzoic acid, *p*-coumaric, ferulic and caffeic acid. Syringic and vanillic acid were not further metabolized, but the concentration of protocatechuic acid slightly decreased after 24 h incubation.	[54]
Isolated cyanidin-3-glucoside, cyanidin-3-rutinoside, malvidin-3-glucoside, delphinidin-3-glucoside, petunidin-3-glucoside and peonidin-3-glucoside	Anthocyanins were incubated with human microbiota	Anthocyanin glucosides were almost completely degraded within the first hour of incubation. Cyanidin-3-rutinoside was initially degraded into cyanidin-3-glucoside. Cyanidin and protochatecuic acid were the main metabolites from cyanidin-3-glucoside, but other metabolites could not be identified.	[53]
Gallic acid, malvidin-glucoside and enocianin	The compounds were incubated for 24 h under large intestine conditions in a batch-culture fermentation system with human microflora from healthy volunteers	Anthocyanins were almost completely degraded within the first 4 h of incubation, whereas gallic acid was stable within the firs 5 h. Syringic acid was the main metabolite from malvidin-3-glucoside, reaching a maximum concentration after 4 h of incubation. Gallic acids and pyrogallol were also identified. Gallic, syringic and *p*-coumaric acids were identified in the media with enocianin. Anthocyanins increased the content of *Bifidobacterium* spp. and *Lactobacillus-Enterococcus* spp.	[57]
Individual cyanidin-3-glucoside, delphinidin-3-glucoside, and malvidin-3-glucoside	Anthocyanins encapsulated with β-cyclodextrin were incubated with gut bacteria from fecal samples of healthy participants	Ferulic, gallic, and syringic acid were the main metabolites of cyanidin-3-glucoside, delphinidin-3-glucoside, and malvidin-3-glucoside, respectively. Gallic acid was also found in malvidin-3-glucoside reactions. β-cyclodextrin delayed anthocyanin degradation. Malvidin-3-glucoside increase the growth of the domain bacteria (EUBmix probe), cyanidin-3-glucoside, and delphinidin-3-glucoside inhibited the growth of proteolytic bacterium *C. histolyticum.*	[55]
Cyanidin-3-glucoside, malvidin-3-glucoside, cyanidin-3,5,-diglucoside, malvidin3,5-diglucoside, cyanidin-3-rutinoside, peonidin-3-glcoside	Anthocyanins were incubated with caecum of freshly slaughtered pigs	Metabolites were detected after 20 min of incubation. They found phloroglucinol aldehyde, protochatechuic acid, syringic acid and vanillic acid. Bacteria also degraded the phenolic acids. However, these phenolic acids were also observed in sterilized media at pH 6.4. The authors suggested a high degradation of anthocyanins into chalcones.	[58]

### 2.4. Premise IV. Few Anthocyanin Metabolites Have Been Accurately Identified in Biological Samples Due to Technical Limitations

Sample preparation previous to anthocyanin analysis largely influences the properties of these compounds. It is recommended that the sample must always be protected from high temperatures and light, as some degradative reactions of anthocyanins are endothermic, and light also degrades anthocyanins [85]. Special attention must also be paid to the pH of the sample and solvents to be used for sample preparation and analysis, as stated above [23]. Overall, the alteration sample’s pH should be avoided as much as possible; however, this alteration is commonly required to complete the analysis of anthocyanins. This adjustment and the anthocyanin analysis must be carried out quickly, especially when the anthocyanins are in citrate and phosphate buffers commonly used to create medium mimicking biological fluids, as buffers are used to exert stabilizing effects on pH and this effect varies over time, affecting anthocyanin structure. By the way, the pH of the sample and any solvent to be mixed with it (e.g., mobile phase for the chromatographic analysis) must be the same in order to avoid changes in anthocyanin structure and absorbance and, consequently, affecting the reliability in identification and quantification of these compounds. Additionally, the chromatographic analysis must be performed immediately after sample collection, monitoring all possible anthocyanin metabolites (flavylium cations, chalcones, hemiacetales, quinonoidal bases, and final anthocyanin metabolites) simultaneously at their maximum wavelengths (510–520, 320–340 and 280 nm). Unfortunately, these good practices for anthocyanin analysis have not been used in many cases, leading to the unreliable identification and quantification of anthocyanin metabolites.

The evaluation of the stability and identity of anthocyanins and their metabolites in physiological fluids or media mimicking physiological conditions has traditionally involved the acidification of samples to revert the modification of anthocyanin structure and generate the most stable form of anthocyanins, the flavyllium cation [20,21,30,32,44,45,47,48,51,52,65]. This analytical strategy allows the transformation of colorless anthocyanin forms to their colored counterparts, which can be studied using common UV-Vis detectors/spectrophotometers. However, acidification leads to the conversion of anthocyanin with different optical properties, and in some cases, the generation of the flavyllium cation by acidification is incomplete or impossible. The low concentration of intact anthocyanins found in blood and tissues may result from an incomplete transformation of chalcone or hemiacetal forms to the flavylium cation during sample preparation for chemical analysis. Talavera et al. [44] studied the intestinal stability of purified anthocyanins by in situ perfusion (37 °C, pH 6.6) on mice intestines after 0, 25, and 45 min of incubation and did not find degradation rates greater than 9.3%, with delphinidin glycosides and cyanidin-3-glucoside being the least and more stable structures, respectively. However, the anthocyanins were quantified after the acidification of samples with 240 mmol/L HCl. Acidification favored the reconstitution of flavylium cation, but it probably caused an underestimation of anthocyanin degradation. Woodward et al. [37] reported high losses of anthocyanins after complete concentration/evaporation of extracts from *In vitro* digestions obtained by solid phase extraction and acidic washes (0.5% HCl). The sudden increase in the concentration of acids after solvent evaporation causes anthocyanin degradation [86]. Trifluoroacetic acid has been preferred for sample acidification due to its relatively low boiling point, which reduces dramatic changes in its content after evaporation. Other simple treatments, such as freezing, may degrade some anthocyanin metabolites, making the rapid analysis of samples important [47]. Overall, these analytical limitations, especially those related to the control of pH, have collectively led to the unreliable identification and quantification of anthocyanin metabolites and, consequently, to the unreliable determination of their contribution to human health.

The acidification step is not needed if MS^n^ or NMR analyses are employed. Some studies have employed MS^2^ or MS^3^ to identify anthocyanin metabolites [23,44,45,49,50,51,52,53,54,55,56,57,58,59,60,67]. Although MS^n^ is a powerful tool for identifying metabolites of many compounds, the extraction and sample handling are important for correctly analyzing anthocyanins. Using this technique, Sadilova et al. [60] identified chalcone forms of pelargonidin-3-glucoside and cyanidin-glucoside-xyloside in extracts from strawberry and elderberry fruit adjusted at pH 3.5. The molecular weight of these chalcones included the additional mass of a water molecule compared with that of the original anthocyanin due to the conversion of the flavylium cation to a carbinol pseudobase. The samples were immediately processed in that study, but, unfortunately, the extracts were not subjected to digestion conditions. A disadvantage of MS^n^ analysis is that the parent anthocyanin and its chalcones show the same molecular ion, making the differentiation among carbinol pseudobase and *cis*- and *trans*-chalcones impossible. Additionally, the site for substitution of glucuronic or sulfuronic acids in the anthocyanin structure cannot be determined using only MS^n^ analysis. Another limitation of MS^n^ analysis is that for the identification of colorless and small metabolites (phloroglucinol aldehyde and phenolic acids), the low limit of the *m/z* range must be reduced up to 200, with this increasing the difficulty to identify such compounds [60]. Kamonpatana et al. [30] found a cyanidin chalcone using HPLC-TOF-analysis, but they did not provide the fragmentation pattern for this anthocyanin metabolite. TOF-MS provides a good resolution and sensitivity for profiling intact precursor ions. Recently, Victoria-Campos et al. [23] identified several chalcones by UPLC-MS-MS and anthocyanin aglycones in digestion reactions of pure individual anthocyanins without acidification of the sample and concluded that the exclusion of this analytical step facilitated the identification of anthocyanin isoforms.

NMR represents another important analytical tool for analyzing the structure of anthocyanins and their metabolites. Santos et al. [87] showed the characterization of two hemiacetals and the cis- and trans-chalcone forms of malvidin in solutions at pH from 0.3 to 4. Jordheim et al. [88] could characterize two hemiacetals forms of anthocyanins combining 1D and 2D NMR thanks to improvements in this technique, such as the development of high-field magnets and cryoprobete technology. Some studies have shown the characterization of chalcone precursors of other flavonoids [29]. However, the low concentration of anthocyanin metabolites in body tissues and fluids makes the NMR analysis challenging, as was reported by Kay et al. [43], who could not determine the glucuronide position in methylated cyanidin metabolites in urine and blood samples. The analysis of anthocyanins and their metabolites in biological samples should be performed by combining separation techniques along UV-Vis, MS^n^, and MNR techniques to obtain more reliable data on anthocyanin structure as a function of the physiological media. Unfortunately, studies including all these analyses for biological fluids recovered from individuals supplemented with pure anthocyanins are very scarce. This type of study represents the correct way to elucidate the metabolism of anthocyanins and the beneficial effects of their metabolites.

## 3. Conclusions

Anthocyanin’s structure is highly altered in the DT due to pH changes and intestinal microflora. This alteration’s extent depends on anthocyanin structure, including the type of anthocyanidin and glycosylation and the number and position of their oxygenated substituents. Unfortunately, few studies have been performed in this regard using pure and semi-purified anthocyanins. This kind of study allows for the accurate identification of anthocyanin metabolites as they eliminate the interference of compounds naturally contained in the test foods identified as anthocyanin metabolites or compounds that can serve as precursors of compounds identified as anthocyanin metabolites. The identification of anthocyanin metabolites in biological samples must also be performed without the acidification of samples to revert the modification of anthocyanin structure and generate the most stable form of anthocyanins (flavylium cation), as this regeneration is generally incomplete and changes the optical properties of anthocyanin metabolites. The use of MS and NMR is encouraged for the accurate identification of anthocyanin metabolites.

The chemical characteristics of anthocyanins and the evidence from the scarce literature about the anthocyanin forms in equilibria and their degradation products indicate that anthocyanins are not accumulated in the body as flavylium cations, the most studied anthocyanin form. Glucuronides, methylated derivatives, chalcones, phenolic acids, and phloroglucinol aldehyde are the most common anthocyanin metabolites in the human body. These anthocyanin metabolites seem to exert the same beneficial effects attributed to intact anthocyanins, including cardioprotective, anticancer, antidiabetic, anti-inflammatory, and neuroprotective effects. These effects must be confirmed by using anthocyanin metabolites rather than anthocyanins.

## Figures and Tables

**Table 1:** Health benefits of anthocyanin consumption according to clinical trials and epidemiological studies.

Model	Dose	Results	Reference
Cardiometabolic Health			
Data for older adults (≥50 years) from the Australian Health Survey (2011–12) (*n* = 3327)	Estimated by a 24 h diet recall	Significant inverse correlation between anthocyanin intake and systolic blood pressure (β = −0.04, *r*^2^ = 0.01; β = −0.01, *r*^2^ = 0.01)	[2]
Overweight and obese subjects (IMC ≥ 25 Kg/m^2^; 32.2 ± 4.6 years) consuming daily tart cherry juice	Placebo vs. 240 mL of tart cherry juice (9.9–23.7 mg of anthocyanins) consumed daily for 4 weeks	Participants in the experimental group (tart cherry juice consumers) had significant lower levels of the chemokine monocyte chemoattractant protein-1 (MCP-1), of the cytokine tumor necrosis factor-α (TNF-α) and of the erythrocyte sedimentation rate than placebo group (*p* < 0.05)	[3]
Clinically healthy subjects (*n* = 35) with normal weight and overweight/obese (23–59 years) consuming daily red orange juice	Daily consumption of 750 mL of red orange juice for 8 weeks	The supplementation significantly reduced the levels of total cholesterol (from 7.4 to 13.3% less), LDL-cholesterol (from 11.9 to 15.2% less), C-reactive protein (from 14.3 to 40.7% less) and the diastolic blood pressure (from 1.4 to 5.1% less). It increased the antioxidant capacity (54.5–62%) (*p* < 0.05) in both healthy and overweight/obese groups	[5]
Dyslipidemic adults (*n* = 93) treated with capsules of bilberry and blackcurrant anthocyanins	Placebo vs. 40, 80, 160 and 320 mg of anthocyanins/day for 12 weeks	A dose–response reduction in collagen-induced platelet aggregation (−7.05%), platelet reactive oxygen species levels (−14.6%) and an increase in mitochondrial membrane potential (7.4%). There was a reduction in the activated glycoprotein GPIIBIIIa (−8.25%) in the 80 mg/day group (*p* < 0.05)	[7]
Dyslipidemic adults (*n* = 176) supplemented with purified anthocyanins from bilberries and blackcurrants	Placebo vs. 40, 80 and 320 mg anthocyanins/day for 12 weeks	The highest dose of anthocyanins (320 mg/day) caused significant changes in cholesterol efflux capacity (CEC) (from −0.11% up to 0.25%), HDL cholesterol (from −0.06 up to 0.043 mmol/L) and apolipoprotein A-I (Apo A-I) (from −0.01 up to 0.06 g/L) (*p* < 0.05) after 12 weeks.	[8]
Dyslipidemic adults (*n* = 169) supplemented with different doses of anthocyanins from blackcurrant and bilberry	Placebo vs. anthocyanin supplementation (40, 80 or 320 mg of anthocyanins/day) during 12 weeks	There was a dose-dependent reduction in the serum inflammatory cytokines IL-6 (up to 40% less), TNF-α (up to 21% less), and MDA (up to 20% lower levels) and the urine oxidative stress biomarkers 8-iso-PGF_2α_ (up to 37% less) and 8-OHdG (up to 36% less) (*p* < 0.05) after 12 weeks.	[11]
Antidiabetic effects			
Healthy and non-healthy (metabolic syndrome disease) subjects (25–75 years; *n* = 35) supplemented with capsules of wild Norwegiant bilberries and blackcurrant extracts	Daily consumption of capsules (320 mg of extract/day) during 4 weeks	Non-healthy participants showed significant reductions in fasting blood glucose (−15.7%), cholesterol (−18.2%), LDL cholesterol (−33.4%), triglycerides (−28.4%), and high-sensitivity C-reactive protein (−36.3%) (*p* < 0.05). There was also a significant increase in expression of superoxide dismutase (47%) and PPAR-γ. There was an inhibition of proinflammatory genes related with the NF-kB pathway: TNF-α (−15–28%), IL-6 (−13.6–16.1%), IL-1A (−12.9–21.5%), PCAM-1 (−15–17.5%) and COX-2 (−26–27%) (*p* < 0.05) in both groups.	[1]
Healthy and non-healthy subjects (risk and diagnosed with type 2 diabetes; *n* = 40) supplemented with capsules of bilberry and black currant anthocyanins	320 mg of anthocyanins/day during 4 weeks	Fasting blood glucose (−14.8%), LDL cholesterol (−22.1%) and uric acid (−16.0%) (*p* < 0.05) decreased in participants at risk of type 2 diabetes. The inflammation biomarkers IL-6, IL-8 and TNF-α significantly decreased in participants diagnosed with type 2 diabetes (*p* < 0.05)	[4]
Adults diagnosed with type 2 diabetes (45–75 years; *n* = 52) supplemented with freeze-dried blueberries	11 g twice a day of freeze-dried blueberries during 8 weeks (261.8 mg of anthocyanins)	Significant reductions in glycated hemoglobin (−1.4%), fructosamine (−5.3%), alanine transaminase (−2.5%), aspartate transaminase (−1.5%) and triglycerides (−3.5%) (*p* < 0.05)	[6]
Adults with elevated fasting glucose (*n* = 121) supplemented with capsules of bilberry and blackcurrant anthocyanins	Placebo vs. 320 mg of anthocyanins/day during 12 weeks	There were significant reductions in fasting glucose (−0.4 mmol/L), 2 h C-peptide (−1.02 ng/mL) and 3 h area under the curve of C-peptide (−2.09) and a significant increase in insulin-like growth factor binding protein-4 (IGFBP-4) fragments (+8.33 ng/mL) (*p* < 0.05)	[9]
Subjects with prediabetes or newly diagnosed diabetes (*n* = 160) treated with purified anthocyanins from bilberries and blackcurrants	Placebo vs. 320 mg of anthocynins/day during 12 weeks	There were significant reductions in serum adiponectin (−0.51 μg/mL), and apoliprotein B (−0.1 g/L) and an increase in liprotein A-1 (0.15 g/L) (*p* < 0.05). Fasting glucose (−0.5 mmol/L) decreased in subjects with diabetes (*p* < 0.05).	[10]
Neuroprotection			
Subjects with mild cognitive impairment or stable non-obstructive coronary disease (≥50 years; *n* = 47) supplemented with capsules of bilberry and blackcurrant anthocyanins	160 mg of anthocynins/day for 16 weeks	Monocyte chemoattractant protein (MCP-1), fasting glucose, total cholesterol, and triglycerides decreased significantly. Memory improvements were also observed.	[12]
Older adults (67.5 years; *n* = 26) supplemented with a blueberry concentrate	Placebo vs. blueberry concentrate (387 mg of anthocyanins/day) for 12 weeks	The blueberry concentrate increased the activity of brain regions related with cognitive function (Broadmann areas 4, 6, 10, 21, 40, 44, 45 and precuneous, anterior cingulate and insula/thalamus) (*p* < 0.001) and improved the perfusion in parietal (5.0%) and occipital lobes (8.0%) (*p* < 0.05)	[13]
Older adults (60–75 years; *n* = 37) supplemented with freeze-dried blueberry	Placebo vs. 12 g of lyophilized blueberry/day during 90 days (230.4 mg of anthocynins)	There was a significant reduction in switch cost on a task-switching test and lower errors for the California Verbal Learning test (number of word recalled) (*p* < 0.05). There were also improvements in their balance when standing with closed eyes	[14]
Older adults (65–80 years; *n* = 122 supplemented with wild berry extracts	Placebo vs. 500 (WBP500) or 100 (WBP 1000) mg of wild berry powder or a commercial wild berry extract 111 mg (WBE111)	After 3 months of treatment, WBE111 improved word recognition following a 20 min delay (5.41%) (*p*< 0.05). WBE111 also decreased the systolic blood pressure after 3 and 6 months of treatment (*p* < 0.05)	[15]
Cancer prevention			
A case (*n* = 923)—control (*n* = 1846) study in a Korean population. Participants answered a semi-quantitative food frequency questionnaire for the estimation of flavonoid intake	Quartiles consumption of anthocyanidins (≥29.7 vs. 18.9 < 29.7, 11.4 < 18.9 and <11.4 mg/day) and other flavonoids related to the risk of colorectal cancer	Higher anthocyanidin intake was associated with a lower risk of colorectal cancer as compared with the lowest intake (48% lower risk, *p* < 0.001). Flavonols and flavan-3-ols were more associated with low risk of colorectal cancer (80% and 62% lower risk, respectively, *p* < 0.001). The flavonol intake was strongly associated with carriers of the CC homozygous variant than of the T allele carriers in rectal cancer.	[16]
A case (*n* = 1522) -control *(n* = 1547) study in a Chinese population about the relation between flavonoid consumption and breast cancer risk	Quartiles of consumption of anthocyanidins and other flavonoids related to the risk of breast cancer	There was an inverse association between consumption of total flavonoids, anthocyanidins, proanthocyanidins, flavanones, flavones, flavonols, and isoflavones and the risk of breast cancer	[17]
A case–control study in an Italian population. Participants answered a food-frequency questionnaire for the estimation of flavonoids (*n* = 1225 and 728 of colon and rectal cancers; and *n* = 4154 of healthy population)	Quintile consumption of anthocyanidins (5.3, 11.5, 19.4, and 31.7 mg/day) and other flavonoids related to the risk of colorectal cancer	Higher anthocyanidin intake decreased the risk of colorectal cancer about 33% as compared with the lowest consumption (*p* < 0.001). Higher consumption of isoflavones, flavones, and flavonols was significantly associated with lower risk of colorectal cancer (from 22% up to 36% lower risk, as compared with the lowest consumption).	[18]
A prospective study within the European Prospective Investigation into Cancer and Nutrition (EPIC) study in subjects from 10 European countries (*n* = 477,312)	Subjects were followed for up to 11 years and evaluated for the association between dietary flavonoid consumption and the risk of colon cancer	The consumption of anthocyanins, flava-3-ols monomers, theaflavins, and flavonols were associated with lower risk of colorectal cancer in former smokers, but not in either never or current smokers	[19]

## Data Availability

No new data were created or analyzed in this study.

## References

[1] Aboonabi A., Aboonabi A. (2020). Anthocyanins reduce inflammation and improve glucose and lipid metabolism associated with inhibiting nuclear factor-kappaB activation and increasing PPAR-γ gene expression in metabolic syndrome subjects. Free Radic. Biol. Med..

[2] Igwe E.O., Charlton K.E., Probst Y.C. (2019). Usual dietary anthocyanin intake, sources and their association with blood pressure in a representative sample of Australian adults. J. Hum. Nutr. Diet..

[3] Martin K.R., Burrell L., Bopp J. (2018). Authentic tart cherry juice reduces markers of inflammation in overweight and obese subjects: A randomized, crossover pilot study. Food Funct..

[4] Nikbakht E., Singh I., Vider J., Williams L.T., Vugic L., Gaiz A., Kundur A.R., Colson N. (2021). Potential of anthocyanin as an anti-inflammatory agent: A human clinical trial on type 2 diabetic, diabetic at-risk and healthy adults. Inflamm. Res..

[5] Silveira J.Q., Dourado G.K., Cesar T.B. (2015). Red-fleshed sweet orange juice improves the risk factors for metabolic syndrome. Int. J. Food Sci. Nutr..

[6] Stote K.S., Wilson M.M., Hallenbeck D., Thomas K., Rourke J.M., Sweeney M.I., Gottschall-Pass K.T., Gosmanov A.R. (2020). Effect of blueberry consumption on cardiometabolic health parameters in men with type 2 diabetes: An 8-week, double-blind, randomized, placebo-controlled trial. Curr. Dev. Nutr..

[7] Tian Z., Li K., Fan D., Zhao Y., Gao X., Ma X., Xu L., Shi Y., Ya F., Zou J. (2021). Dose-dependent effects of anthocyanin supplementation on platelet function in subjects with dyslipidemia: A randomized clinical trial. EBioMedicine.

[8] Xu Z., Xie J., Zhang H., Pang J., Li Q., Wang X., Xu H., Sun X., Zhao H., Yang Y. (2021). Anthocyanin supplementation at different doses improves cholesterol efflux capacity in subjects with dyslipidemia—A randomized controlled trial. Eur. J. Clin. Nutr..

[9] Yang L., Liu Z., Ling W., Wang L., Wang C., Ma J., Peng X., Chen J. (2020). Effect of anthocyanins supplementation on serum IGFBP-4 fragments and glycemic control in patients with fasting hyperglycemia: A randomized controlled trial. Diabetes Metab. Syndr. Obes..

[10] Yang L., Ling W., Qiu Y., Liu Y., Wang L., Yang J., Wang C., Ma J. (2020). Anthocyanins increase serum adiponectin in newly diagnosed diabetes but not in prediabetes: A randomized controlled trial. Nutr. Metab..

[11] Zhang H., Xu Z., Zhao H., Wang X., Pang J., Li Q., Yang Y., Ling W. (2020). Anthocyanin supplementation improves anti-oxidative and anti-inflammatory capacity in a dose–response manner in subjects with dyslipidemia. Redox Biol..

[12] Bergland A.K., Soennesyn H., Dalen I., Rodriguez-Mateos A., Berge R.K., Giil L.M., Rajendran L., Siow R., Tassotti M., Larsen A.I. (2019). Effects of anthocyanin supplementation on serum lipids, glucose, markers of inflammation and cognition in adults with increased risk of dementia–a pilot study. Front. Genet..

[13] Bowtell J.L., Aboo-Bakkar Z., Conway M.E., Adlam A.L.R., Fulford J. (2017). Enhanced task-related brain activation and resting perfusion in healthy older adults after chronic blueberry supplementation. Appl. Physiol. Nutr. Metab..

[14] Miller M.G., Hamilton D.A., Joseph J.A., Shukitt-Hale B. (2018). Dietary blueberry improves cognition among older adults in a randomized, double-blind, placebo-controlled trial. Eur. J. Nutr..

[15] Whyte A.R., Cheng N., Fromentin E., Williams C.M. (2018). A randomized, double-blinded, placebo-controlled study to compare the safety and efficacy of low dose enhanced wild blueberry powder and wild blueberry extract (ThinkBlue™) in maintenance of episodic and working memory in older adults. Nutrients.

[16] Cho Y.A., Lee J., Oh J.H., Chang H.J., Sohn D.K., Shin A., Kim J. (2017). Dietary flavonoids, CYP1A1 genetic variants, and the risk of colorectal cancer in a Korean population. Sci. Rep..

[17] Feng X.L., Ho S.C., Mo X.F., Lin F.Y., Zhang N.Q., Luo H., Zhang C.X. (2020). Association between flavonoids, flavonoid subclasses intake and breast cancer risk: A case-control study in China. Eur. J. Cancer Prev..

[18] Rossi M., Negri E., Talamini R., Bosetti C., Parpinel M., Gnagnarella P., La Vecchia C. (2006). Flavonoids and colorectal cancer in Italy. Cancer Epidemiol. Biomark. Prev..

[19] Zamora-Ros R., Barupal D.K., Rothwell J.A., Jenab M., Fedirko V., Romieu I., Scalbert A. (2017). Dietary flavonoid intake and colorectal cancer risk in the European prospective investigation into cancer and nutrition (EPIC) cohort. Int. J. Cancer.

[20] Felgines C., Talavera S., Texier O., Gil-Izquierdo A., Lamaison J.L., Remesy C. (2005). Blackberry anthocyanins are mainly recovered from urine as methylated and glucuronidated conjugates in humans. J. Agric. Food Chem..

[21] Lapidot T., Harel S., Granit R., Kanner J. (1998). Bioavailability of red wine anthocyanins as detected in human urine. J. Agric. Food Chem..

[22] Rosales T.K.O., da Silva F.F.A., Rivera A.G., dos Santos S.N., Bustos D., Morales-Quintana L.A., Santos H.A., Soares Bernardes E., Fabi J.P. (2024). A study of the oral bioavailability and biodistribution increase of nanoencapsulation-driven delivering radiolabeled anthocyanins. Food Res. Int..

[23] Victoria-Campos C.I., Ornelas-Paz J.J., Rocha-Guzmán N.E., Gallegos-Infante J.A., Failla M.L., Pérez-Martínez J.D., Rios-Velasco C., Ibarra-Junquera V. (2022). Gastrointestinal metabolism and bioaccessibility of selected anthocyanins isolated from commonly consumed fruits. Food Chem..

[24] Mueller D., Jung K., Winter M., Rogoll D., Melcher R., Richling E. (2017). Human intervention study to investigate the intestinal accessibility and bioavailability of anthocyanins from bilberries. Food Chem..

[25] Boto-Ordóñez M., Urpi-Sarda M., Queipo-Ortuño M.I., Tulipani S., Tinahones F.J., Andres-Lacueva C. (2014). High levels of Bifidobacteria are associated with increased levels of anthocyanin microbial metabolites: A randomized clinical trial. Food Funct..

[26] Stalmach A., Edwards C.A., Wightman J.D., Crozier A. (2013). Colonic catabolism of dietary phenolic and polyphenolic compounds from Concord grape juice. Food Funct..

[27] Brouillard R., Dubois J.E. (1977). Mechanism of the structural transformations of anthocyanins in acidic media. J. Am. Chem. Soc..

[28] Preston N.W., Timberlake C.F. (1981). Separation of anthocyanin chalcones by high-performance liquid chromatography. J. Chromatogr. A.

[29] Cabrera M., Lavaggi M.L., Croce F., Celano L., Thomson L., Fernández M., Pintos C., Raymondo S., Bollati M., Monge A. (2010). Identification of chalcones as *in vivo* liver monofunctional phase II enzymes inducers. Bioorg. Med. Chem..

[30] Kamonpatana K., Giusti M.M., Chitchumroonchokchai C., Moreno-Cruz M., Riedl K.M., Kumar P., Failla M.L. (2012). Susceptibility of anthocyanins to *ex vivo* degradation in human saliva. Food Chem..

[31] Samota M.K., Yadav D.K., Koli P., Kaur M., Kaur M., Rani H., Selvan S.S., Mahala P., Tripathi K., Kumar S. (2024). Exploring natural chalcones: Innovative extraction techniques, bioactivities and health potential. Sustain. Food. Technol..

[32] Kalt W., Liu Y., McDonald J.E., Vinqvist-Tymchuk M.R., Fillmore S.A.E. (2014). Anthocyanin metabolites are abundant and persistent in human urine. J. Agric. Food Chem..

[33] Brouillard R., Markakis P. (1982). Chemical structure of anthocyanins. Anthocyanins as Food Colors.

[34] Wang J., Zhao Y., Sun B., Yang Y., Wang S., Feng Z., Li J. (2024). The structure of anthocyanins and the copigmentation by common micromolecular copigments: A review. Food Res. Int..

[35] Torskangerpoll K., Andersen Ø.M. (2005). Colour stability of anthocyanins in aqueous solutions at various pH values. Food Chem..

[36] Furtado P., Figueiredo P., das Neves H.C., Pina P. (1993). Photochemical and thermal degradation of anthocyanins. J. Photochem. Photobiol. A. Chem..

[37] Woodward G., Kroon P., Cassidy A., Kay C. (2009). Anthocyanin stability and recovery: Implications for the analysis of clinical and experimental Samples. J. Agric. Food Chem..

[38] Wu X., Cao G., Prior R. (2002). Absorption and metabolism of anthocyanins in elderly women after consumption of elderberry of blueberry. J. Nutr..

[39] Fornasaro S., Ziberna L., Gasperotti M., Tramer F., Vrhovšek U., Mattivi F., Passamonti S. (2016). Determination of cyanidin 3-glucoside in rat brain, liver and kidneys by UPLC/MS-MS and its application to a short-term pharmacokinetic study. Sci. Rep..

[40] Chen Y., Chen H., Zhang W., Ding Y., Zhao T., Zhang M., Mao G., Feng W., Wu X., Yang L. (2019). Bioaccessibility and biotransformation of anthocyanin monomers following *In vitro* simulated gastric-intestinal digestion and *in vivo* metabolism in rats. Food Funct..

[41] Gui H., Sun L., Liu R., Si X., Li D., Wang Y., Shu C., Sun X., Jiang Q., Qiao Y. (2023). Current knowledge of anthocyanin metabolism in the digestive tract: Absorption, distribution, degradation, and interconversion. Crit. Rev. Food Sci. Nutr..

[42] Felgines C., Texier O., Garcin P., Besson C., Lamaison J.L., Scalbert A. (2009). Tissue distribution of anthocyanins in rats fed a blackberry anthocyanin-enriched diet. Mol. Nutr. Food Res..

[43] Kay C.D., Mazza G.J., Holub B.J. (2005). Anthocyanins exist in the circulation primarily as metabolites in adult men. J. Nutr..

[44] Talavera S., Felgines C., Texier O., Besson C., Manach C., Lamaison J.L., Rémésy C. (2004). Anthocyanins are efficiently absorbed from the small intestine in rats. J. Nutr..

[45] Talavera S., Felgines C., Texier O., Besson C., Gil-Izquierdo A., Lamaison J.L., Rémésy C. (2005). Anthocyanin metabolism in rats and their distribution to digestive area, kidney, and brain. J. Agric. Food Chem..

[46] Płatosz N., Bączek N., Topolska J., Szawara-Nowak D., Skipor J., Milewski S., Wiczkowski W. (2021). Chokeberry anthocyanins and their metabolites ability to cross the blood-cerebrospinal fluid barrier. Food Chem..

[47] Felgines C., Talavera S., Gonthier M.P., Texier O., Scalber A., Lamaison J.L., Rémésy C. (2003). Strawberry anthocyanins are recovered in urine as glucuro- and sulfoconjugates in humans. J. Nutr..

[48] Aqil F., Vadhanam M.V., Jeyabalan J., Cai J., Singh I.P., Gupta R.C. (2014). Detection of anthocyanins/anthocyanidins in animal tissues. J. Agric. Food Chem..

[49] Azzini E., Vitaglione P., Intorre F., Napolitano A., Durazzo A., Foddai M.S., Fumagalli A., Catasta G., Rossi L., Venneria E. (2010). Bioavailability of strawberry antioxidants in human subjects. Br. J. Nutr..

[50] Vitaglione P., Donnarumma G., Napolitano A., Galvano F., Gallo A., Scalfi L., Fogliano V. (2007). Protocatechuic acid is the major human metabolite of cyanidin-glucosides. J. Nutr..

[51] Kay C.D., Kroon P.A., Cassidy A. (2009). The bioactivity of dietary anthocyanins is likely to be mediated by their degradation products. Mol. Nutr. Food Res..

[52] Nurmi T., Mursu J., Heinonen M., Nurmi A., Hiltunen R., Voutilainen S. (2009). Metabolism of berry anthocyanins to phenolic acids in humans. J. Agric. Food Chem..

[53] Aura A.M., Martin-Lopez P., O’Leary K.A., Williamson G., Oksman-Caldentey K.M., Poutanen K., Santos-Buelga C. (2005). *In vitro* metabolism of anthocyanins by human gut microflora. Eur. J. Nutr..

[54] Fleschhut J., Kratzer F., Rechkemmer G., Kulling S.E. (2006). Stability and biotransformation of various dietary anthocyanins *In vitro*. Eur. J. Nutr..

[55] Flores G., del Castillo M.L.R., Costabile A., Klee A., Guergoletto K.B., Gibson G.R. (2015). *In vitro* fermentation of anthocyanins encapsulated with cyclodextrins: Release, metabolism and influence on gut microbiota growth. J. Funct. Foods..

[56] Guergoletto K.B., Costabile A., Flores G., Garcia S., Gibson G.R. (2016). *In vitro* fermentation of juçara pulp (Euterpe edulis) by human colonic microbiota. Food Chem..

[57] Hidalgo M., Oruna-Concha M.J., Kolida S., Walton G.E., Kallithraka S., Spencer J.P., Pascual-Teresa S. (2012). Metabolism of anthocyanins by human gut microflora and their influence on gut bacterial growth. J. Agric. Food Chem..

[58] Keppler K., Humpf H.U. (2005). Metabolism of anthocyanins and their phenolic degradation products by the intestinal microflora. Bioorg. Med. Chem..

[59] Sánchez-Patán F., Cueva C., Monagas M., Walton G.E., Gibson M., Quintanilla-López J.E., Lebron-Aguilar R., Martin-Álvarez P.J., Victoria Moreno-Arribas M., Bartolomé B. (2012). *In vitro* fermentation of a red wine extract by human gut microbiota: Changes in microbial groups and formation of phenolic metabolites. J. Agric. Food Chem..

[60] Sadilova E., Carle R., Stintzing F.C. (2007). Thermal degradation of anthocyanins and its impact on color and *In vitro* antioxidant capacity. Mol. Nutr. Food Res..

[61] Gamel T.H., Wright A.J., Tucker A.J., Pickard M., Rabalski I., Podgorski M., Di Ilio N., O’Brien C., Abdel-Aal E.S.M. (2019). Absorption and metabolites of anthocyanins and phenolic acids after consumption of purple wheat crackers and bars by healthy adults. J. Cereal Sci..

[62] Chen T.Y., Kritchevsky J., Hargett K., Feller K., Klobusnik R., Song B.J., Cooper B., Jouni Z., Ferruzzi M.G., Janle E.M. (2015). Plasma bioavailability and regional brain distribution of polyphenols from apple/grape seed and bilberry extracts in a young swine model. Mol. Nutr. Food Res..

[63] Czank C., Cassidy A., Zhang Q., Morrison D.J., Preston T., Kroon P.A., Botting N.P., Kay C.D. (2013). Human metabolism and elimination of the anthocyanin, cyanidin-3-glucoside: A 13C-tracer study. Am. J. Clin. Nutr..

[64] Kirakosyan A., Seymour E.M., Wolforth J., McNish R., Kaufman P.B., Bolling S.F. (2015). Tissue bioavailability of anthocyanins from whole tart cherry in healthy rats. Food Chem..

[65] Kalt W., Blumberg J.B., McDonald J.E., Vinqvist-Tymchuk M.R., Fillmore S.A., Graf B.A., O’Leary J.M., Milbury P.E. (2008). Identification of anthocyanins in the liver, eye, and brain of blueberry-fed pigs. J. Agric. Food Chem..

[66] Tavares L., Figueira I., Macedo D., McDougall G.J., Leitão M.C., Vieira H.L.A., Sterwart D., Alves P.M., Ferreira R.B., Santos C.N. (2012). Neuroprotective effect of blackberry (*Rubus* sp.) polyphenols is potentiated after simulated gastrointestinal digestion. Food Chem..

[67] Mostafa H., Behrendt I., Meroño T., González-Domínguez R., Fasshauer M., Rudloff S., Andres-Lacueva C., Kuntz S. (2023). Plasma anthocyanins and their metabolites reduce *In vitro* migration of pancreatic cancer cells, PANC-1, in a FAK-and NF-kB dependent manner: Results from the ATTACH-study a randomized, controlled, crossover trial in healthy subjects. Biomed. Pharmacother..

[68] Kuntz S., Kunz C., Rudloff S. (2017). Inhibition of pancreatic cancer cell migration by plasma anthocyanins isolated from healthy volunteers receiving an anthocyanin-rich berry juice. Eur. J. Nutr..

[69] Augusti P.R., Quatrin A., Mello R., Bochi V.C., Rodrigues E., Prazeres I.D., Macedo A.C., Oliveira-Alves S.C., Emanuelli T., Bronze M.R. (2021). Antiproliferative effect of colonic fermented phenolic compounds from jaboticaba (*Myrciaria trunciflora*) fruit peel in a 3D cell model of colorectal cancer. Molecules.

[70] Dong A., Lin C.W., Echeveste C.E., Huang Y.W., Oshima K., Yearsley M., Chen X., Yu J., Wang L.S. (2022). Protocatechuic acid, a gut bacterial metabolite of black raspberries, inhibits adenoma development and alters gut microbiome profiles in *Apc*^min/+^ mice. J. Cancer Prev..

[71] Peiffer D.S., Zimmerman N.P., Wang L.S., Ransom B.W., Carmella S.G., Kuo C.T., Siddiqui J., Chen J.H., Oshima K., Huang Y.W. (2014). Chemoprevention of esophageal cancer with black raspberries, their component anthocyanins, and a major anthocyanin metabolite, protocatechuic acid. Cancer Prev. Res..

[72] Li H., Zheng T., Lian F., Xu T., Yin W., Jiang Y. (2022). Anthocyanin-rich blueberry extracts and anthocyanin metabolite protocatechuic acid promote autophagy-lysosomal pathway and alleviate neurons damage in *in vivo* and *In vitro* models of Alzheimer’s disease. Nutrition.

[73] Forester S.C., Choy Y.Y., Waterhouse A.L., Oteiza P.I. (2014). The anthocyanin metabolites gallic acid, 3-O-methylgallic acid, and 2, 4,6-trihydroxybenzaldehyde decrease human colon cancer cell viability by regulating pro-oncogenic signals. Mol. Carcinog..

[74] Sankaranarayanan R., Valiveti C.K., Kumar D.R., Kesharwani S.S., Seefeldt T., Scaria J., Tummala H., Bhat G.J. (2019). The flavonoid metabolite 2, 4, 6-trihydroxybenzoic acid is a CDK inhibitor and an anti-proliferative agent: A potential role in cancer prevention. Cancers.

[75] Min S.W., Ryu S.N., Kim D.H. (2010). Anti-inflammatory effects of black rice, cyanidin-3-O-β-d-glycoside, and its metabolites, cyanidin and protocatechuic acid. Int. Immunopharmacol..

[76] Forester S.C., Waterhouse A.L. (2010). Gut metabolites of anthocyanins, gallic acid, 3-O-methylgallic acid, and 2, 4, 6-trihydroxybenzaldehyde, inhibit cell proliferation of Caco-2 cells. J. Agric. Food Chem..

[77] Grace M.H., Ribnicky D.M., Kuhn P., Poulev A., Logendra S., Yousef G.G., Raskin I., Lila M.A. (2009). Hypoglycemic activity of a novel anthocyanin-rich formulation from lowbush blueberry, *Vaccinium angustifolium* Aiton. Phytomedicine.

[78] Chua A.W., Hay H.S., Rajendran P., Shanmugam M.K., Li F., Bist P., Shanmugam M.K., Li F., Bist P., Koay E.S. (2010). Butein downregulates chemokine receptor CXCR4 expression and function through suppression of NF-κB activation in breast and pancreatic tumor cells. Biochem. Pharmacol..

[79] Mallery S.R., Budendorf D.E., Larsen M.P., Pei P., Tong M., Holpuch A.S., Larsen P.E., Stoner G.D., Fields H.W., Chan K.K. (2011). Effects of human oral mucosal tissue, saliva, and oral microflora on intraoral metabolism and bioactivation of black raspberry anthocyanins. Cancer Prev. Res..

[80] Jang H.S., Kook S.H., Son Y.O., Kim J.G., Jeon Y.M., Jang Y.S., Choi K.C., Kim J., Han S.K., Lee K.Y. (2005). Flavonoids purified from Rhus verniciflua Stokes actively inhibit cell growth and induce apoptosis in human osteosarcoma cells. Biochim. Biophys. Acta.

[81] Lee J.C., Lee K.Y., Kim J., Na C.S., Jung N.C., Chung G.H., Jang Y.S. (2004). Extract from Rhus verniciflua Stokes is capable of inhibiting the growth of human lymphoma cells. Food. Chem. Toxicol..

[82] Stanley M.P.P., Rajakumar S., Dhanasekar K. (2011). Protective effects of vanillic acid on electrocardiogram, lipid peroxidation, antioxidants, proinflammatory markers and histopathology in isoproterenol induced cardiotoxic rats. Eur. J. Pharmacol..

[83] Milenkovic D., Krga I. (2024). Anthocyanin metabolites maintain brain endothelial cell function and permeability through a multigenomic mode of action. Curr. Dev. Nutr..

[84] Tan J., Li Y., Hou D.X., Wu S. (2019). The effects and mechanisms of cyanidin-3-glucoside and its phenolic metabolites in maintaining intestinal integrity. Antioxidants.

[85] Enaru B., Drețcanu G., Pop T.D., Stǎnilǎ A., Diaconeasa Z. (2021). Anthocyanins: Factors affecting their stability and degradation. Antioxidants.

[86] He J., Giusti M.M. (2011). High-purity isolation of anthocyanins mixtures from fruits and vegetables–A novel solid-phase extraction method using mixed mode cation-exchange chromatography. J. Chromatogr. A.

[87] Santos H., Turner D.L., Lima J.C., Figueiredo P., Pina F., Macanita A. (1993). Elucidation of the multiple equilibra of malvidin in aqueous solution by one- and two-dimensional NMR. Phytochemistry.

[88] Jordheim M., Fossen T., Andersen O.M. (2006). Characterization of hemiacetal forms of anthocyanidin 3-*O*-β-glycopyranosides. J. Agric. Food Chem..

